# Implementing the 3Rs in Laboratory Animal Research—From Theory to Practice

**DOI:** 10.3390/ani13193063

**Published:** 2023-09-29

**Authors:** Garikoitz Azkona

**Affiliations:** Department of Basic Psychological Processes and Their Development, Euskal Herriko Unibertsitatea (UPV/EHU), Tolosa Hiribidea 70, 20018 Donostia, Spain; garikoitz.azkona@ehu.eus

The regulatory framework for the use of animals in research in many countries is based on the 3Rs: replacement, reduction, and refinement [1]. These principles state that if it is necessary to use animals in experiments, researchers should make every effort to replace them with non-sentient alternatives, reduce their numbers to a minimum, and refine experiments and housing conditions to minimize pain and distress as much as possible. Thus, the 3Rs concept serves both as a framework designed to minimize animal use and suffering (harm to the animal) and as a means to support high-quality science and translation (benefit to society) [2].

This Special Issue compiles the latest research results and advances relevant to the 3Rs. A total of 23 papers have been published: 12 research articles, 1 commentary, 2 communications, 1 concept paper, 5 reviews, and 2 systematic reviews. The contributions are listed below.

Most of the published articles and communications have focused on the third R: refinement. In terms of husbandry, it has been observed that male CD1 mice raised together with environmental enrichment in well-ventilated cages showed fewer signs of stress (1). Conversely, single-housed mice exhibited changes in the immune–endocrine system (2). Concerning experimental processes, the use of clicker training improved compliance in the catwalk test (3), and acclimation and saphenous vein puncture for blood collection reduced stress in C57Bl/6J mice (4). Two articles explore the use of imaging tools to reduce animal numbers and improve their welfare, employing Positron Emission Tomography (PET) to track animals throughout their lives (5) and camera-based respiration monitoring, which reduces animal handling (6). In the same vein, a gelatin-based voluntary ingestion protocol is proposed to administer drugs (7). Pérez-Martin et al. (8) describe a refined stereotaxic neurosurgery technique for long-term intracerebroventricular device implantation in rodents. A score sheet is proposed to evaluate the animal welfare of the type 2 diabetes rat model induced by streptozotocin following fructose consumption (9). Two papers focus on replacement: the use of organoids to evaluate cellular therapies (10) and a new in vitro assay to determine the biological activity of insulins (11). Peruga and collaborators (12) ponder whether current animal models are useful in researching how female hormones influence orthodontic biomechanics. The fourth R, rehoming, has been a positive experience using golden hamsters after their use in SARS-CoV-2 vaccine research (13).

In their commentary, Verderio et al. (14) provide an overview of the current status of the 3Rs and emphasize the need for bioinformaticians to achieve high standards of animal research. The review articles of this Special Issue have focused on the importance of animal models in biomedical research (15), the most widely used techniques to implement the 3Rs in experimental liver research (16), the adverse impacts of sex bias on science and animal welfare (17), the gaps and challenges in primate pain management (18), and ultrasound-guided surgery as a refinement tool in oncology research (19). Regarding systematic reviews, one focuses on the possible causes and solutions to aggression between grouped male mice (20), and the other summarizes published advances in the refinement protocols made by European Union-based research groups in the last 10 years (21). Finally, in his concept paper, David B. Morton (22) proposes a mathematical model to analyze measurement data to determine the degree of harm (or severity) incurred by an animal during research. Likewise, De Vleeschauwer et al. (23) developed a severity classification for all procedures performed in two Belgian academic biomedical institutions.

Overall, this Special Issue presents a range of perspectives on current research in implementing the 3Rs, from practical applications to theoretical frameworks, all with the shared aim of enhancing the welfare of laboratory animals.

## List of Contributions

1. Lee, G.-H.; Kim, K.; Jo, W. Stress Evaluation of Mouse Husbandry Environments for Improving Laboratory Animal Welfare. *Animals* **2023**, *13*, 249. https://doi.org/10.3390/ani13020249.
2. Ortega-Saez, I.; Díez-Solinska, A.; Grífols, R.; Martí, C.; Zamora, C.; Muñoz-Culla, M.; Vegas, O.; Azkona, G. Individualized Housing Modifies the Immune–Endocrine System in CD1 Adult Male Mice. *Animals* **2023**, *13*, 1026. https://doi.org/10.3390/ani13061026.
3. Dickmann, J.; Gonzalez-Uarquin, F.; Reichel, S.; Pichl, D.; Radyushkin, K.; Baumgart, J.; Baumgart, N. Clicker Training Mice for Improved Compliance in the Catwalk Test. *Animals* **2022**, *12*, 3545. https://doi.org/10.3390/ani12243545.
4. Marin, N.; Moragon, A.; Gil, D.; Garcia-Garcia, F.; Bisbal, V. Acclimation and Blood Sampling: Effects on Stress Markers in C57Bl/6J Mice. *Animals* **2023**, *13*, 2816. https://doi.org/10.3390/ani13182816.
5. Palumbo, G.; Kunze, L.H.; Oos, R.; Wind-Mark, K.; Lindner, S.; von Ungern-Sternberg, B.; Bartenstein, P.; Ziegler, S.; Brendel, M. Longitudinal Studies on Alzheimer Disease Mouse Models with Multiple Tracer PET/CT: Application of Reduction and Refinement Principles in Daily Practice to Safeguard Animal Welfare during Progressive Aging. *Animals* **2023**, *13*, 1812. https://doi.org/10.3390/ani13111812.
6. Breuer, L.; Mösch, L.; Kunczik, J.; Buchecker, V.; Potschka, H.; Czaplik, M.; Pereira, C.B. Camera-Based Respiration Monitoring of Unconstrained Rodents. *Animals* **2023**, *13*, 1901. https://doi.org/10.3390/ani13121901.
7. Ruvira, S.; Rodríguez-Rodríguez, P.; Cañas, S.; Ramiro-Cortijo, D.; Aguilera, Y.; Muñoz-Valverde, D.; Arribas, S.M. Evaluation of Parameters Which Influence Voluntary Ingestion of Supplements in Rats. *Animals* **2023**, *13*, 1827. https://doi.org/10.3390/ani13111827.
8. Pérez-Martín, E.; Coto-Vilcapoma, A.; Castilla-Silgado, J.; Rodríguez-Cañón, M.; Prado, C.; Álvarez, G.; Álvarez-Vega, M.A.; Fernández-García, B.; Menéndez-González, M.; Tomás-Zapico, C. Refining Stereotaxic Neurosurgery Techniques and Welfare Assessment for Long-Term Intracerebroventricular Device Implantation in Rodents. *Animals* **2023**, *13*, 2627. https://doi.org/10.3390/ani13162627.
9. Silva-Reis, R.; Faustino-Rocha, A.I.; Silva, J.; Valada, A.; Azevedo, T.; Anjos, L.; Gonçalves, L.; Pinto, M.d.L.; Ferreira, R.; Silva, A.M.S.; et al. Studying and Analyzing Humane Endpoints in the Fructose-Fed and Streptozotocin-Injected Rat Model of Diabetes. *Animals* **2023**, *13*, 1397. https://doi.org/10.3390/ani13081397.
10. García-Delgado, A.B.; Campos-Cuerva, R.; Rosell-Valle, C.; Martin-López, M.; Casado, C.; Ferrari, D.; Márquez-Rivas, J.; Sánchez-Pernaute, R.; Fernández-Muñoz, B. Brain Organoids to Evaluate Cellular Therapies. *Animals* **2022**, *12*, 3150. https://doi.org/10.3390/ani12223150.
11. Rüggeberg, S.; Wanglin, A.; Demirel, Ö.; Hack, R.; Niederhaus, B.; Bidlingmaier, B.; Blumrich, M.; Usener, D. Progress towards the Replacement of the Rabbit Blood Sugar Test for the Quantitative Determination of the Biological Activity of Insulins (USP <121>) with an In Vitro Assay. *Animals* **2023**, *13*, 2953. https://doi.org/10.3390/ani13182953.
12. Peruga, M.; Kawala, B.; Sarul, M.; Kotowicz, J.; Lis, J. Are Currently Selected Laboratory Animals Useful in the Research of How Female Hormones Influence Orthodontic Biomechanics? *Animals* **2023**, *13*, 629. https://doi.org/10.3390/ani13040629.
13. Štrbenc, M.; Kuhar, U.; Lainšček, D.; Orehek, S.; Slavec, B.; Krapež, U.; Malovrh, T.; Majdič, G. Rehoming and Other Refinements and Replacement in Procedures Using Golden Hamsters in SARS-CoV-2 Vaccine Research. *Animals* **2023**, *13*, 2616. https://doi.org/10.3390/ani13162616.
14. Verderio, P.; Lecchi, M.; Ciniselli, C.M.; Shishmani, B.; Apolone, G.; Manenti, G. 3Rs Principle and Legislative Decrees to Achieve High Standard of Animal Research. *Animals* **2023**, *13*, 277. https://doi.org/10.3390/ani13020277.
15. Domínguez-Oliva, A.; Hernández-Ávalos, I.; Martínez-Burnes, J.; Olmos-Hernández, A.; Verduzco-Mendoza, A.; Mota-Rojas, D. The Importance of Animal Models in Biomedical Research: Current Insights and Applications. *Animals* **2023**, *13*, 1223. https://doi.org/10.3390/ani13071223.
16. Martinez-Lopez, S.; Angel-Gomis, E.; Sanchez-Ardid, E.; Pastor-Campos, A.; Picó, J.; Gomez-Hurtado, I. The 3Rs in Experimental Liver Disease. *Animals* **2023**, *13*, 2357. https://doi.org/10.3390/ani13142357.
17. Nunamaker, E.A.; Turner, P.V. Unmasking the Adverse Impacts of Sex Bias on Science and Research Animal Welfare. *Animals* **2023**, *13*, 2792. https://doi.org/10.3390/ani13172792.
18. Paterson, E.A.; Turner, P.V. Challenges with Assessing and Treating Pain in Research Primates: A Focused Survey and Literature Review. *Animals* **2022**, *12*, 2304. https://doi.org/10.3390/ani12172304.
19. Camara Serrano, J.A. Ultrasound Guided Surgery as a Refinement Tool in Oncology Research. *Animals* **2022**, *12*, 3445. https://doi.org/10.3390/ani12233445.
20. Weber, E.M.; Zidar, J.; Ewaldsson, B.; Askevik, K.; Udén, E.; Svensk, E.; Törnqvist, E. Aggression in Group-Housed Male Mice: A Systematic Review. *Animals* **2023**, *13*, 143. https://doi.org/10.3390/ani13010143.
21. Díez-Solinska, A.; Vegas, O.; Azkona, G. Refinement in the European Union: A Systematic Review. *Animals* **2022**, *12*, 3263. https://doi.org/10.3390/ani12233263.
22. Morton, D.B. A Model Framework for the Estimation of Animal ‘Suffering’: Its Use in Predicting and Retrospectively Assessing the Impact of Experiments on Animals. *Animals* **2023**, *13*, 800. https://doi.org/10.3390/ani13050800.
23. De Vleeschauwer, S.; Lambaerts, K.; Hernot, S.; Debusschere, K. Severity Classification of Laboratory Animal Procedures in Two Belgian Academic Institutions. *Animals* **2023**, *13*, 2581. https://doi.org/10.3390/ani13162581.
